# Evolution of High Tooth Replacement Rates in Sauropod Dinosaurs

**DOI:** 10.1371/journal.pone.0069235

**Published:** 2013-07-17

**Authors:** Michael D. D’Emic, John A. Whitlock, Kathlyn M. Smith, Daniel C. Fisher, Jeffrey A. Wilson

**Affiliations:** 1 Anatomical Sciences Department, Stony Brook University, Health Sciences Center, School of Medicine, Stony Brook, New York, United States of America; 2 Science and Mathematics Department, Mount Aloysius College, Cresson, Pennsylvania, United States of America; 3 Department of Geology & Geography and Georgia Southern University Museum, Georgia Southern University, Statesboro, Georgia, United States of America; 4 Museum of Paleontology and Department of Earth & Environmental Sciences, University of Michigan, Ann Arbor, Michigan, United States of America; Monash University, Australia

## Abstract

**Background:**

Tooth replacement rate can be calculated in extinct animals by counting incremental lines of deposition in tooth dentin. Calculating this rate in several taxa allows for the study of the evolution of tooth replacement rate. Sauropod dinosaurs, the largest terrestrial animals that ever evolved, exhibited a diversity of tooth sizes and shapes, but little is known about their tooth replacement rates.

**Methodology/Principal Findings:**

We present tooth replacement rate, formation time, crown volume, total dentition volume, and enamel thickness for two coexisting but distantly related and morphologically disparate sauropod dinosaurs *Camarasaurus* and *Diplodocus*. Individual tooth formation time was determined by counting daily incremental lines in dentin. Tooth replacement rate is calculated as the difference between the number of days recorded in successive replacement teeth. Each tooth family in *Camarasaurus* has a maximum of three replacement teeth, whereas each *Diplodocus* tooth family has up to five. Tooth formation times are about 1.7 times longer in *Camarasaurus* than in *Diplodocus* (315 vs. 185 days). Average tooth replacement rate in *Camarasaurus* is about one tooth every 62 days versus about one tooth every 35 days in *Diplodocus*. Despite slower tooth replacement rates in *Camarasaurus*, the volumetric rate of *Camarasaurus* tooth replacement is 10 times faster than in *Diplodocus* because of its substantially greater tooth volumes. A novel method to estimate replacement rate was developed and applied to several other sauropodomorphs that we were not able to thin section.

**Conclusions/Significance:**

Differences in tooth replacement rate among sauropodomorphs likely reflect disparate feeding strategies and/or food choices, which would have facilitated the coexistence of these gigantic herbivores in one ecosystem. Early neosauropods are characterized by high tooth replacement rates (despite their large tooth size), and derived titanosaurs and diplodocoids independently evolved the highest known tooth replacement rates among archosaurs.

## Introduction

Large or complex dentitions generally experience attrition through abrasion against food, substrates, or other teeth. In mammals, food intake and tooth use tend to increase with body size, so larger animals tend to exhibit increased tooth wear [1]. During their nearly 300-million-year evolutionary history [2], vertebrate herbivores evolved numerous mechanisms to cope with increased tooth wear, including changes in the mechanical properties of tooth tissues [3], [4], increases in the number of teeth that are functional at one time [5], [6], continuous tooth growth and eruption throughout the life of the animal [7], increases in the number of tooth-bearing bones, changes in crown volume and/or shape [8]–[11], and increased tooth replacement rate [12], [13].

Sauropod dinosaurs achieved the largest adult body sizes of any terrestrial herbivore, and so would have required a large food supply and high levels of tooth use and tooth wear regardless of their inferred physiology [14]–[16]. Evolutionary responses to high tooth wear in sauropods – including changes in tooth volume and tooth replacement rate – are first recorded shortly after their divergence from sauropodomorph ancestors. Early Jurassic sauropods increased tooth size, but decreases in tooth size characterized some later-appearing lineages [10]. The volume of the tooth crown has a demonstrated relationship to its formation time and expected use-life [12], [13] and is inversely related to the number of teeth that can be held at each tooth position [10]. High tooth replacement rates were calculated in one sauropod with up to ten small teeth packed at each tooth position [6], but neither the relationship between tooth volume and replacement rate nor the relationship between these parameters and the overall rate of replacement of the total functional dentition have been studied for other sauropods.

Here, we measure tooth formation time, replacement rate, crown volume, and enamel thickness in sectioned teeth of *Camarasaurus* and *Diplodocus*, two neosauropod dinosaurs from the Late Jurassic Morrison Formation of North America. The largest exemplars of these two genera are similar in body mass (e.g., femur length ca. 1.8 m, sum of femoral and humeral circumference ca. 1.3 m; MDD unpublished data), but they belong to distantly related neosauropod clades that differ substantially in skull morphology, body proportions, and inferred feeding ecology [17]–[20]. The rarity of sauropod craniodental materials that can be sacrificed for histological sampling limits the taxonomic scope across which we can measure these features. We explore the distribution of these features more broadly within Sauropodomorpha by developing a method to estimate tooth replacement rates for several taxa that have craniodental material but cannot be sampled histologically.

## Materials and Methods

Permission was received to access the relevant specimens from museum collections managers. Specimens were loaned from the Yale Peabody Museum, Utah Museum of Natural History, Staatliches Museum für Naturkunde, and Iziko South African Museum. Computed tomography (CT) images were acquired at the Canton Health Center, University of Michigan, using a General Electric Lightspeed Pro 8 CT scanner, GE Medical systems, Milwaukee, Wisconsin. CT slices were taken using 140 Kv and 325 mA, with 1.250 mm thick slices and 0.625 mm overlap. Incremental lines were counted in thin section. Each tooth was mechanically removed from the jaws. Specimens were embedded in epoxy resin, cut longitudinally on a Buehler Isomet saw with a diamond wafering blade, mounted on a glass slide, cut to a thick section, and hand-sanded and polished until incremental lines were visible. Thin sections were photographed using a Spot CCD camera (Spot Insight 11.2 Color Mosaic, Diagnostic Instruments) mounted on a Nikon SMZ 1500 microscope. Increments were counted in ImageJ [21], [22] using the IncMeas v1.11 plug-in [23].

**Figure 1:**
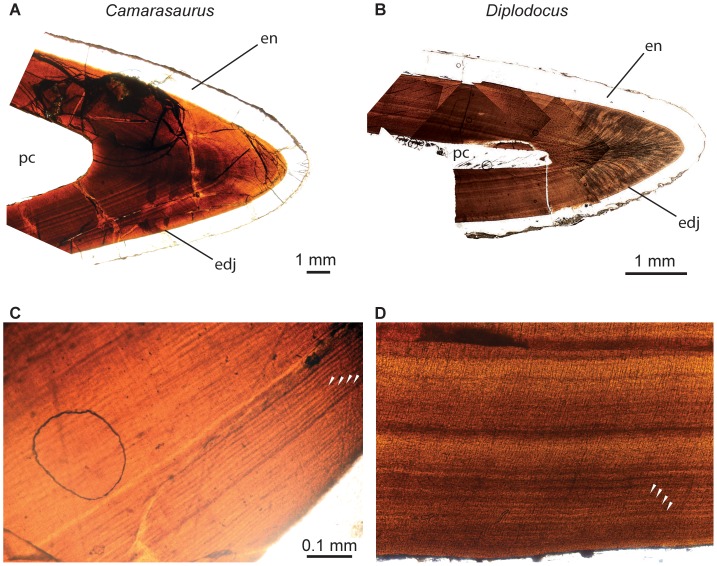
Dental histology of the sauropod dinosaurs *Camarasaurus* and *Diplodocus*. Thin sections of *Camarasaurus* (**A**, **C**) and *Diplodocus* (**B**, **D**) premaxillary teeth showing incremental lines of von Ebner (white arrowheads) in dentin. Teeth are oriented with their long axis horizontal and the occlusal surface directed to the right. **A** shows the tip of tooth 3iii of *Camarasaurus*, and **B** shows the tip of tooth 4iv of *Diplodocus*. **C** and **D** are enlarged images of one ‘limb’ of tooth 3ii and 4iii, respectively. Abbreviations: **edj**, enamel-dentin junction; **en**, enamel; **pc**, pulp cavity. [planned for page width].

Tooth formation time and replacement rate in *Diplodocus* and *Camarasaurus* were measured by counting incremental lines of von Ebner (Fig. 1), which have been shown to represent daily fronts of dentin deposition in several groups of extant amniotes [12], [13], [24]–[26]. We define tooth replacement rate to be the time required to replace one tooth in a given alveolus. This rate is sometimes expressed in days, with the unit numerator implicit. Replacement rate was calculated by subtracting tooth formation times for successive teeth within one family, following Erickson [12], [13]. Recently, Scheyer and Moser [27] questioned the identification of incremental lines of von Ebner in sauropods, suggesting that they could represent longer-period increments (e.g., Andresen lines). We examined our thin sections and did not find smaller increments between the lines spaced ca. 15 microns apart in areas where preservation seems excellent, so we interpret these lines as daily fronts of deposition. The ca. 15-micron spacing of incremental lines of von Ebner observed in *Diplodocus* and *Camarasaurus* is close to the mean value observed in labelling studies of adult *Alligator* [13].

Enamel thickness was measured in ImageJ on photographs of thin sections. Thickness was measured perpendicular to the enamel-dentin junction at seven locations around the tooth crown (three labial, three lingual, and one apical). Fewer measurements were made on teeth for which enamel was chipped or missing in certain locations. Labial and lingual measurements were taken at roughly evenly spaced locations along the apicobasal axis (one near the tooth tip or apex, one near the mid-length of the crown, and one near the crown-root junction). Enamel thickness varies around the tooth crown, so comparison of labial and lingual thicknesses for each tooth was based on the average of three labial measurements and the average of three lingual measurements. An overall average of all measurements taken on a single tooth was also calculated. Raw enamel measurements are presented in Supporting Information (Raw Data S1).

Volumes of both the entire tooth and the crown (i.e., the part of the tooth covered in enamel, including the pulp cavity) were measured by water displacement via suspension three times and averaged (see Raw Data S1) [28]. Total erupted tooth volume (the sum of the volumes of all ‘fresh’ [functional but unworn] teeth in the jaw) and crown volume (the sum of the crown volumes of all fresh teeth in the jaw) were estimated for *Camarasaurus* and *Diplodocus*. Tooth crowns are similar in volume for adjacent teeth throughout and among jaw elements for all tooth positions except for the last few in these species. For each species, the antepenultimate and penultimate tooth crown volumes were estimated as 75% of the average measured tooth crown volume, and the last tooth position was estimated as 50% of the average measured tooth crown volume. In contrast to total crown volumes, total functional tooth volumes were more complicated to estimate because the alternating pattern of tooth replacement in these species yields tooth roots of substantially different size in adjacent teeth. The average of total functional tooth volumes for two large teeth was used as the functional individual tooth volume. As with crowns, total functional tooth volumes for the antepenultimate, penultimate, and ultimate tooth positions were estimated as 75%, 75%, and 50% of the volume of the largest teeth, respectively. Finally, in *Diplodocus*, dentary teeth are about 10% smaller in volume than premaxillary or maxillary teeth [29], so estimates of the volumes of dentary teeth and crowns were adjusted accordingly.

In many cases, destructive sampling of a specimen was not possible. For these taxa, we developed a non-invasive approach for estimating replacement rate, based on use of *Camarasaurus* as a model for taxa with broad-crowned teeth (*Patagosaurus*, *Mamenchisaurus*) and *Diplodocus* as a model for taxa with narrow-crowned teeth (*Nigersaurus*, Río Negro titanosaur). Both models were used for estimation of replacement rate in *Massospondylus*, which has an intermediate crown breadth. Tooth length was measured for teeth of *Camarasaurus* and *Diplodocus* for which ages were already known via counts of incremental lines of von Ebner. For each genus, regression of tooth formation time on tooth length generated an equation that was used to estimate tooth formation time in teeth that were not sampled histologically.

We estimated volumetric tooth replacement rate (the time required to replace the total dentition-in-use) by dividing total erupted tooth volume by average tooth replacement rate. We made a similar estimate using only tooth crown volumes (volumetric crown replacement rate). We make the assumption that tooth replacement rate was constant throughout and among jaw elements due to similarities in the shape, number of replacement teeth, and depth of alveoli in all but the distal-most few teeth in each jaw element. Our simplification would tend to inflate the volumetric replacement rates, but would affect each species similarly, thus keeping results for each comparable to one another.

## Results

Histology-based tooth replacement rates and estimated replacement rates are summarized in Tables 1–2. The dentary of the basal sauropodomorph *Plateosaurus* did not show any replacement teeth in CT images, so no further analysis was undertaken. CT scans of a maxilla and dentary of the basal sauropodomorph *Massospondylus* revealed only a single replacement tooth (in one alveolus of the dentary). Although we did thin section teeth of *Massospondylus*, tooth replacement rate for that genus was estimated because incremental lines were poorly preserved.

**Table 1:** Tooth formation time (days) and replacement rate (1 tooth/X days) in *Diplodocus* (YPM 4677) and *Camarasaurus* (UMNH 5527).

*Diplodocus* tooth family							
		1	2	3		4	
tooth position	i	–	187	–		183	
	ii	176	–	178		145	
	iii	141	–	144		113	
	iv	–	–	110		–	
	v	–	–	67		–	
**average replacement rate**		**35**	**–**	**37**		**35**	
***Camarasaurus*** **tooth family**							
		1	2	3		4	
tooth position	i	–	–	–		315	
	ii	–	–	208		253	
	iii	–	–	–		190	
	iv	–	–	–		130	
**average replacement rate**		–	–		–		**62**

Abbreviations: UMNH, Utah Museum of Natural History, Salt Lake City, USA; YPM, Yale Peabody Museum, New Haven, USA.

**Table 2:** Estimated tooth formation times (ages) and replacement rates in several sauropodomorphs.

taxon	estimated replacement rate (days)
*Massospondylus* (SAM-PK-K39)	17–30
*Patagosaurus* (MPEV-PV 1670)	58
*Mamenchisaurus* (ZDM0083)	98
*Diplodocus* (YPM 4677)	34
*Nigersaurus* (MNN GAD-512)	14
*Camarasaurus* (UMNH 5527)	62
Río Negro specimen (MPCA-79)	20

There is a range for *Massospondylus* tooth replacement rate because estimates were made both using the narrow-crowned and broad-crowned equations. Note the similarity of estimated replacement rates for *Camarasaurus* and *Diplodocus* with histologically obtained rates of 62 and 35 days for these taxa, respectively.

Abbreviations: MNN, Musée National du Niger, Niamey, Niger; MPCA, Museo Provincial Carlos Ameghino, Cipolletti, Argentina; SAM, South African Museum, Ikizo Museums, Cape Town, South Africa; UMNH, Utah Museum of Natural History, Salt Lake City, USA; YPM, Yale Peabody Museum, New Haven, USA; ZDM, Zigong Dinosaur Museum, Zigong, China.

**Figure 2:**
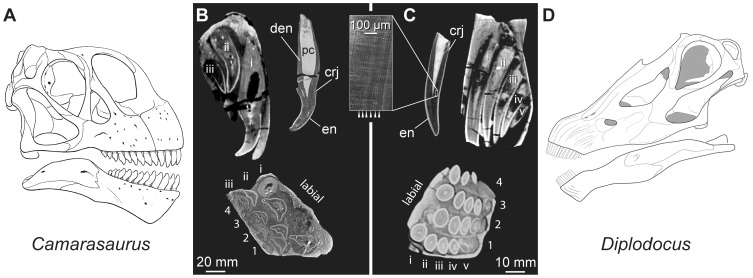
Tooth replacement in the sauropod dinosaurs *Camarasaurus* and *Diplodocus*. Reconstructed skulls (**A**, **D**) and premaxillary teeth (**B**, **C**) of *Camarasaurus* (**A**, **B**) and *Diplodocus* (**C**, **D**). **B** and **C** include CT-generated sagittal and transverse sections of premaxillary alveoli and photographs of thin sections of *Camarasaurus* (UMNH 5527) and *Diplodocus* (YMP 4677). Premaxillae show replacement teeth in each of the four alveoli adjacent to the symphysis labelled by their position along the tooth row (1–4) and their position in the replacement sequence at each tooth position (i–v). Sagittal sections in **B** and **C** were taken at premaxillary tooth position 4 in *Camarasaurus* and premaxillary tooth position 1 in *Diplodocus*. The symphysis faces the bottom of the page in transverse sections. Photographs of thin sections of *Diplodocus* and *Camarasaurus* teeth show enamel (**en**), the pulp cavity (**pc**), daily-deposited incremental lines of von Ebner (arrowheads mark every other line) in the dentin (**den**), and the crown-root junction (**crj**). The 20 mm scale bar is for the premaxilla and tooth images in (**B**); 10 mm scale bar is for premaxilla and tooth images in (**C**). Skull reconstructions are from [19], [46][planned for page width].

CT scans reveal that each premaxillary tooth family of *Camarasaurus* (Fig. 2a, Movies S1 and S2) includes one functional and up to three replacement teeth, whereas each premaxillary tooth family of *Diplodocus* (Fig. 2d, Movie S3) includes up to one functional and five replacement teeth. Incremental lines of von Ebner visible in thin section (Figs. 1–2; Table 1) indicate that each premaxillary tooth of *Camarasaurus* took over ten months to form (ca. 1 tooth/315 days), whereas each *Diplodocus* tooth took only six months to form (ca. 1 tooth/185 days). Average tooth replacement rate in *Camarasaurus* was one tooth per two months (ca. 1 tooth/62 days), whereas it was about one tooth per month in *Diplodocus* (ca. 1 tooth/35 days).

Functional premaxillary teeth of the *Camarasaurus* and *Diplodocus* individuals are 26.5 cm^3^ and 1.7 cm^3^ in volume, respectively. Tooth crowns of the *Camarasaurus* and *Diplodocus* individuals are 15.7 cm^3^ and 1.5 cm^3^ in volume, respectively. We estimate total functional (i.e., erupted) tooth volume across the dentition to be 1,272 cm^3^ in *Camarasaurus* and 69 cm^3^ in *Diplodocus*. When measuring only tooth crowns, these values are 754 cm^3^ and 63 cm^3^, respectively. The volumetric tooth replacement rate was about 10 times greater in *Camarasaurus* (1272 cm^3^/62 days = 20.5 cm^3^/day) than in *Diplodocus* (69 cm^3^/35 days = 2.0 cm^3^/day). The volumetric crown replacement rate was about seven times greater in *Camarasaurus* (754 cm^3^/62 days = 12.2 cm^3^/day) than in *Diplodocus* (63 cm^3^/35 days = 1.8 cm^3^/day).

Premaxillary tooth crowns of *Camarasaurus* individuals have ca. 1.0 mm-thick enamel on both the labial and lingual surfaces of the teeth; in contrast, the enamel of *Diplodocus* is thinner overall (ca. 0.5 mm) and is slightly asymmetrical, with the enamel on the labial face of the tooth about 125–150% the enamel thickness on the lingual face (Figs. 1–2, Table 3).

**Table 3:** Summary of enamel thickness (mm) in *Diplodocus* (YPM 4677) and *Camarasaurus* (UMNH 5527) at different tooth developmental stages (i–v).

*Diplodocus*	i	ii	iii	iv	v
average thickness	0.45	0.45	0.31	0.23	0.08
labial/lingual	1.25	1.41	1.53	1.19	1.12

*Camarasaurus*	i	ii	iii	iv	
average thickness	0.97	0.62	0.38	0.12	
labial/lingual	0.99	0.93	0.86	1.23	

Each value shown is an average of teeth from more than one alveolus at similar stages of development (e.g., ‘ii’ is an average of values for tooth position 2ii, 3ii, 4ii, etc.). See Raw Data S1.

Our non-invasive approach to estimating tooth replacement rate allowed us to evaluate a broader spectrum of sauropodomorphs. Tooth length and age are strongly related in both *Camarasaurus* and *Diplodocus* (R^2^>0.95), but the equations describing these relationships differed between the taxa (see Raw Data S1). We evaluated the performance of our estimation method by estimating tooth replacement rate in *Camarasaurus* and *Diplodocus*, taxa for which replacement rate is known. For a given tooth and its successor, our method of estimating both formation time and replacement rate was generally accurate to within one week. When successive replacement estimates are averaged for several teeth in a single jaw element, the estimates are off by one day at most. Because we were only able to measure the length of one tooth and its successor for each of the non-histologically sampled taxa (aside from the case of *Nigersaurus*), we expect that our estimates are accurate for those taxa to within one week.

**Figure 3:**
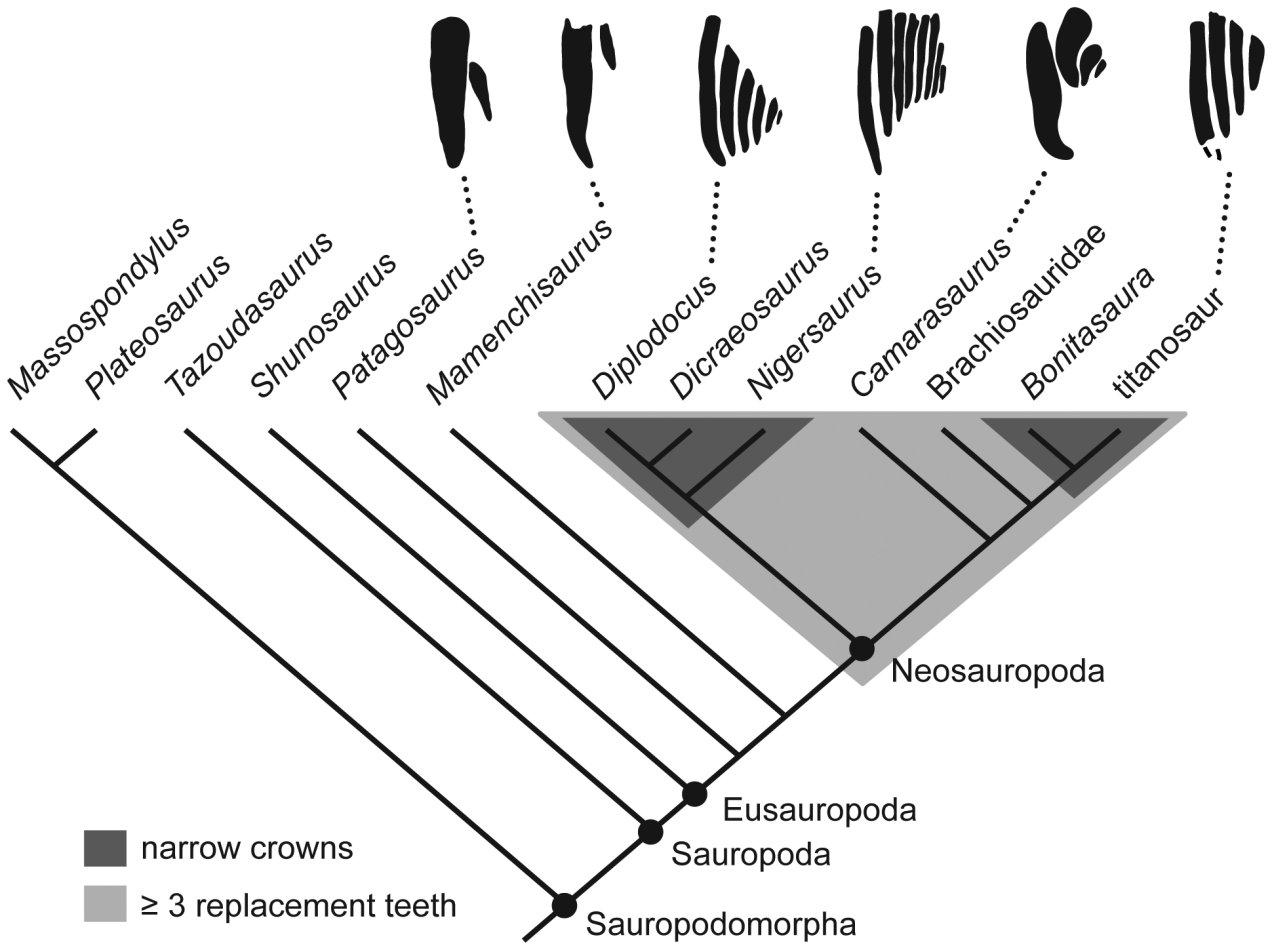
Cladogram of sauropodomorphs showing the optimization of key features related to elevated tooth replacement rates. The light gray field indicates taxa that have at least three replacement teeth at each tooth position; dark gray field encapsulates taxa that have narrow tooth crowns. Silhouettes along the top of the cladogram show the number and size of replacement teeth in one tooth position. These include (from left to right): *Patagosaurus* (MPEF-PV 1670), *Mamenchisaurus* [47], *Diplodocus* (this study), *Nigersaurus* [Sereno, Wilson, Witmer, Whitlock, Maga, Ide and Rowe, unpublished data], *Camarasaurus* (this study), and the Río Negro titanosaur (MPCA-79) [48]. Number of replacement teeth is unknown in Brachiosauridae, but the taxon is optimized to have had at least three. Cladogram based on [30] with the addition of *Tazoudasaurus* [49] and *Bonitasaura* [50]. [planned for column width].

The initial increase in tooth size and crown breadth that occurred near the base of Sauropoda was accompanied by a reduction in tooth replacement rate (as estimated by replacement tooth length). The much smaller teeth of basal sauropodomorphs like *Massospondylus* and *Patagosaurus* formed and replaced faster than did the larger teeth of basal sauropods like *Mamenchisaurus* (Fig. 3; Table 2). Derived broad-crowned taxa (e.g., *Camarasaurus*) exhibited a higher replacement rate than non-neosauropods like *Mamenchisaurus* and matched the rate in the much smaller-toothed *Patagosaurus,* but did not achieve rates as high as those observed in the smaller-toothed *Massospondylus*. Non-neosauropods exhibit a maximum of two replacement teeth per alveolus, whereas neosauropods exhibit three to nine ([6], [30], Fig. 3). Within Neosauropoda, diplodocoids and titanosaurs independently achieved higher tooth replacement rates than basal neosauropods (Fig. 3). The highly specialized diplodocoid *Nigersaurus* is estimated to have replaced each tooth as often as once every 14 days, twice as fast as previous estimates [30 days, [6]] and by far the highest rate for any dinosaur. The discrepancy between our estimate and a previous one for *Nigersaurus* is potentially explainable by the fact that the histology-based replacement rate previously reported for *Nigersaurus* [6] was based on transverse thin sections of teeth. Transverse sections likely yield inaccurate replacement rates due to the limited number of incremental lines of von Ebner exposed in any given transverse plane.

## Discussion

In both *Camarasaurus* and *Diplodocus*, a volume equivalent to approximately one tooth is replaced across the dentition every 1–2 days (20.5 cm^3^/day and 2.0 cm^3^/day, respectively). These taxa are characterized by different styles of forming and replacing dentition: *Camarasaurus* has larger teeth that are replaced less frequently, whereas *Diplodocus* has smaller teeth that are replaced more frequently. Even with *Camarasaurus*’ lower tooth replacement rates, both sauropods exhibit tooth replacement rates on par with or higher than those of non-sauropod dinosaurian herbivores (i.e., hadrosauroid and ceratopsian ornithischians at 50–83 days; Table 4).

**Table 4:** Tooth replacement rates (days) for archosaurs.

taxon	tooth replacement rate (days)
Archosauria	
crocodiliform	105
Dinosauria	
Ornithischia	
* Triceratops*	83
Hadrosauridae	
* Maiasaura*	58
* Edmontosaurus*	50
* Prosaurolophus*	81
Saurischia	
Sauropoda	
* Camarasaurus*	62
* Diplodocus*	35
* Nigersaurus*	14–30
Theropoda	
* Tyrannosaurus*	777
* ‘albertosaur’*	454
* Deinonychus*	290

Data for sauropods are from this study; other data are from [6], [12].

The enamel of *Camarasaurus* is roughly symmetrical labiolingually, in contrast to the slightly asymmetrical enamel of *Diplodocus*. The enamel of the diplodocoid *Nigersaurus* is highly asymmetrical, with enamel on the labial side up to ten times thicker than on the lingual side [5], [6], [31]. Labiolingually asymmetrical enamel appears to characterize several diplodocoids, and extremely asymmetric enamel characterizes *Nigersaurus* or a slightly more inclusive clade [6]. Labiolingually asymmetrical enamel, reduced crown volume, increased replacement rate, and the development of tooth batteries evolved independently in two other dinosaur clades: iguanodontian ornithopods [32] and ceratopsian marginocephalians [33]. The repeated evolution of these features together may represent an adaptation to herbivory at large body size and within the context of polyphyodonty, though several important differences in the evolution of these features exist as well [5].

Sauropods were obligate herbivores, but their antecedents were omnivorous [34]–[37]. The origin and early evolution of sauropods involved increases in tooth volume [10] and body size [38]. Although herbivory and extremely large body size persisted among the vast majority of sauropods, multiple lineages drastically reduced the volume of functional crowns [10]. The repeated independent evolution of narrow crowns suggests that they conferred an adaptive advantage over broad crowns during the second half of sauropod evolution. By the Late Cretaceous, only narrow-crowned sauropod taxa remained [10], [39]. Additionally, following the disappearance of diplodocoids from the fossil record in the early Late Cretaceous, tooth crowns in titanosauriforms decreased in volume and breadth until they were similar in size and shape to those of diplodocoids [10]. Sauropods with broad-crowned teeth (e.g., *Camarasaurus*) evolved tooth replacement rates on par with those of ornithischian herbivores that persisted into the latest Cretaceous, and each *Camarasaurus* tooth was more resistant to wear than smaller teeth by virtue of their larger volume and thicker enamel. Why then did several neosauropod lineages develop narrow-crowned teeth?

One explanation is that fresh teeth are more effective than worn teeth. Replacing a tooth every month reduces the number of excessively worn crowns in the functional dentition, which prolongs contact with opposing teeth and with food. Additionally, although individual teeth were being replaced more frequently, the smaller crown volume results in a lower rate of mineralized tissue production and loss – narrow-crowned taxa had to recoup around 10% of the crown volume of dental tissue that was required in their larger-crowned relatives per tooth replaced. Furthermore, the narrow crowns of *Diplodocus* are made up of a larger proportion of enamel to dentin than the broad crowns of *Camarasaurus*. The advent of narrow-crowned dentition therefore enabled the animal to have many more fresh teeth at any given time, while losing far less mineralized tissue. Furthermore, smaller, more slender teeth would have allowed for smaller tooth roots (Fig. 2) and smaller and lighter cranial bones, resulting in a lighter skull overall. A small head-to-body volume ratio sets sauropods apart from other dinosaurian herbivores [10].

In a finite element analysis of the skull of *Diplodocus*, Young et al. [40] identified high stresses at the bases of teeth that would have been incurred during branch stripping or other feeding strategies. In the context of those results, they interpreted high tooth replacement rates in *Diplodocus* as an adaptation that would have accommodated increased levels of tooth breakage. Aside from concerns that tooth breakage is maladaptive, producing ineffective and infection-prone teeth, we briefly discuss one testable consequence of the Young et al. hypothesis. If *Diplodocus* and other narrow-crowned sauropods experienced tooth breakage as a result of branch stripping or static biting, then the fossil record should bear evidence of such failure. Although the record of cranial remains is sparse for sauropods, it is relatively good for *Diplodocus* and other narrow-crowned sauropods, and we know of no evidence of jaws preserving teeth broken in life.

Rather than being related to high levels of tooth breakage, we propose that increased replacement rates are related to increased wear rates that may have been a consequence of a shift in diet [10], [20]. Some narrow-crowned taxa (e.g., *Diplodocus*) were likely low-browsers [6], [18], [20], [41], [42], a behavior that leads to increased ingestion of abrasive exogenous grit [43]. The sauropod most highly specialized for low browsing, *Nigersaurus*, also has the highest known replacement rate of any dinosaur. In contrast, sauropods with broader tooth crowns and slower replacement rates, such as *Mamenchisaurus* and *Camarasaurus*, are thought to have been mid- to upper-canopy browsers [18], [20], [39], [41], [42], [44], [45], where exogenous grit levels are expected to be lowest.

### Conclusions

Tooth replacement rate, size, and shape data indicate that despite their somewhat stereotyped body plan and large body size, sauropod dinosaurs exhibited varied approaches to feeding. The coexisting but morphologically disparate and distantly related Late Jurassic sauropods *Camarasaurus* and *Diplodocus* differed greatly in their anatomy related to food acquisition: *Camarasaurus* had a large volume of broad-crowned teeth that were replaced relatively slowly, whereas *Diplodocus* had a small volume of narrow-crowned teeth that were replaced very quickly. This variety represents a potential factor that allowed multiple gigantic species such as *Camarasaurus* and *Diplodocus* to partition the same ecosystem. The repeated evolution of narrow-crowned teeth in sauropods appears to have been accompanied by an increase in tooth replacement rate, which would have equipped these forms with less worn teeth over their lifetimes and allowed their skulls to be lighter.

## Supporting Information

Raw Data S1 Microsoft Excel spreadsheet containing tooth volumes, crown volumes, enamel thicknesses, tooth lengths, and estimation method for tooth replacement rate for various sauropodomorphs investigated in this study.
(XLSX)

Movie S1 CT-generated movie of the premaxilla of *Camarasaurus* (UMNH 5527) in mesiodistal view (see separate.mov file)
(MOV)

Movie S2 CT-generated movie of the premaxilla of *Camarasaurus* (UMNH 5527) in apicobasal view (see separate.mov file).
(MOV)

Movie S3 CT-generated movie of the premaxilla of *Diplodocus* (YPM 4677), with bone rendered transparent and teeth opaque (see separate.avi file).
(WMV)
